# Type 1 diabetes mellitus (T1DM) does not affect whole blood responses to alginate-based microspheres despite plasma lipid and glucose differences

**DOI:** 10.1016/j.mtbio.2025.102113

**Published:** 2025-07-18

**Authors:** Kalaiyarasi Vasuthas, Sverre Christian Christiansen, Joachim Sebastian Kjesbu, Liv Ryan, Trygve Andreassen, Geir Slupphaug, Berit L. Strand, Jørgen Stenvik, Anne Mari A. Rokstad

**Affiliations:** a Center of Molecular Inflammation Research (CEMIR), NTNU, Norway; b Department of Clinical and Molecular Medicine, NTNU, Norway; c Department of Endocrinology, St Olav's Hospital, Trondheim University Hospital, Trondheim, Norway; d Department of Biotechnology and Food Science, NTNU, Norway; e Department of Circulation and Medical Imaging, NTNU, Trondheim, Norway; f Central Staff, St. Olavs Hospital HF, Trondheim, Norway; g Clinic of Laboratory Medicine, St Olavs Hospital, Trondheim, Norway; h Clinic of Anaesthesia and Intensive Care, St. Olavs Hospital, Trondheim University Hospital, Trondheim, Norway

**Keywords:** Alginate microspheres, Lipoproteins, Sulfated alginate, Immune response, T1DM, Whole blood model

## Abstract

Encapsulation of insulin-producing cells holds significant therapeutic potential for treating type 1 diabetes (T1DM). The impact of diabetic conditions on host responses is not well understood. This study is the first to compare ex-vivo whole blood responses to alginate microbeads in T1DM subjects versus healthy controls.

Nineteen T1DM and 27 healthy controls were included. Alginate microbeads varying in guluronic acid content (68 % and 47 % G), sulfated alginate/alginate ratios (10/90 and 20/80), or containing poly-L-lysine were characterised regarding activation of coagulation, complement, and inflammatory cytokine release in whole blood. Responses to heat-killed microbes (*E. coli*, *S. pneumoniae*, *M. tuberculosis*, and *C. albicans*) and immune agonists (TLR ligands, T-cell stimulant, and Dectin ligand) were also compared.

NMR spectroscopy identified 40 altered lipoproteins and metabolites in plasma in T1DM vs. controls. However, whole blood responses were strikingly similar, indicating that metabolic alterations in T1DM are not accompanied by differences in inflammatory capacity.

When merging all subjects, PCA clustered microbead responses into three groups of alginates, sulfated alginates, and poly-L-lysine-coated alginate (AP), respectively. Pairwise comparison by multiple t-tests identified significant changes in inflammatory mediators between the main groups; alginate microbeads differentially induced 25 out of 29 mediators compared to AP microbeads and 18 mediators compared to sulfated alginate microbeads. Alginate microbeads with distinct guluronic acid content (68 % vs 47 % G) revealed no significant differences. These findings indicate that the material properties are the most important determinants of the host inflammatory responses in blood, which are not changed in well-controlled T1DM.

## Highlights

• First study: T1DM vs. controls' blood responses to alginate microspheres and immunostimulants.
• NMR spectroscopy identifies 40 altered lipoproteins and metabolites in T1DM vs. controls.
• T1DM and healthy controls show similar blood responses despite altered metabolic profiles.
• Sulfated vs. high G alginate microbeads show changes in 19 out of 29 mediators.
• High G vs. Intermediate alginate microbeads show no difference in 29 parameters.

## Graphical abstract

**Figure:**
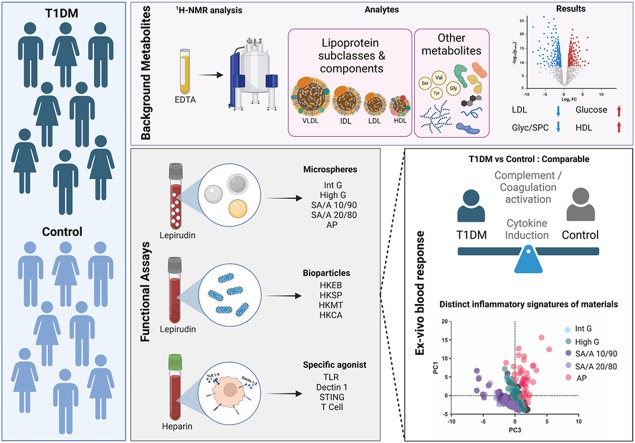

## 1 Introduction

Type 1 diabetes mellitus (T1DM) is a severe autoimmune condition characterised by T cell-mediated destruction of insulin-producing β-cells, leading to hyperglycaemia and ketoacidosis if left untreated [1]. Subcutaneous (SC) insulin delivery lowers blood glucose levels; however, inevitable episodes of hyper- and hypoglycaemia still occur due to slow or insufficient SC insulin uptake, overdosing, and systemic hyperinsulinemia, which may also alter plasma lipid profiles [[2], [3], [4], [5]]. These metabolic imbalances are also apparent in individuals with well-controlled T1DM [3,4], contributing to an elevated risk of cardiovascular disease [6]. While T1DM is currently managed through lifelong SC insulin delivery, recent research aims to develop curative strategies [7,8]. Despite advancements such as continuous glucose monitoring (CGM) and hybrid closed-loop insulin delivery systems, many T1DM patients still experience micro- and macro-vascular complications [1]. Encapsulating insulin-producing cells within microspheres presents a promising controlled-release approach for managing T1DM [9], potentially mitigating glycaemic fluctuations.

Encapsulation of pancreatic islets or stem cell-derived beta cells within alginate microspheres has shown promise in managing blood glucose levels in diabetic mice [[10], [11], [12]], non-human primate macaques [13] and one T1DM patient receiving immunosuppressive [14]. Trials in T1DM patients without immunosuppression have demonstrated safety, although normoglycemia was not consistently achieved [15]. This limitation appears to result from reduced oxygen and nutrient diffusion caused by fibrosis on the microsphere surface, which further impairs encapsulated cell function [16]. Fibrosis is influenced by an inflammatory environment, in which material surface properties play a major role [17,18]. Key factors determining biocompatibility in terms of fibrosis include protein absorption patterns, acute phase protein reactivity [18], monocyte/macrophage responses [19], and lipid deposition profiles [20].

While T1DM is primarily an autoimmune disorder, studies show alterations in innate immune functions relevant to material-host interactions. These include changes in acute-phase proteins [3,[21], [22], [23], [24]], immune cell populations [25,26], systemic lipid composition, and the expression of immune-related proteins. For example, acute-phase proteins like C-reactive protein, α-2-macroglobulin, α-1-antitrypsin, haptoglobin, and fibrinogen are elevated in individuals with poor glycemic control [23,24,27]. Conversely, several complement-related proteins involved in both innate and adaptive immunity are reduced in T1DM [21,22]. T1DM patients have fewer circulatory immune cells, including natural killer cells, dendritic cells, regulatory T cells, and B cells compared to healthy controls [25,28,29]. They also show elevated levels of toll-like receptor (TLR) 2 and 4 on monocytes [30] and elevated levels of circulating TLR ligands [31]. Collectively, these immune dysregulations may affect host responses to biomaterials used in diabetes therapy.

Alginate is a linear polysaccharide composed of 1,4-linked β-D-mannuronic acid (M) and α-L-guluronic acid (G) residues, arranged in blocks of M, G, or alternating MG [32]. In the presence of divalent cations such as calcium, barium, or strontium, alginates form ionically crosslinked hydrogels via junction zones via G-blocks [33]. G-rich alginate microspheres have been evaluated in phase I clinical trials with two T1DM patients [15,34] and in the short-term treatment of acute liver failure in children [35]. While found clinically safe for T1DM, fibrotic overgrowth remains a challenge for long-term function [15,34]. Fibrosis has also been observed upon transplantation of hepatocytes in alginate microbeads for the treatment of acute liver failure [35]. Fibrosis can be reduced by incorporating chemically modified alginate [13,36,37], such as alginate sulfated at the C2 and C3 positions as described by Arlov et al. [38]. This type of modification has previously been shown to reduce fibrosis formation around both empty and cell-containing microspheres [36,39]. The effect of microsphere design on host responses has been further explored using an *ex vivo* human whole-blood model [36,39,40], assessing complement and cytokine activation [41,42], coagulation [43] and plasmin activity [39]. Given the alterations in complement, leukocyte TLRs, and lipid levels in T1DM, it is crucial to determine whether immune responses to engineered materials differ in this condition.

In this study, we investigated the initial immune responses in T1DM and non-diabetic healthy controls using human whole-blood models. This approach integrates coagulation, complement and blood cell communications, thus mimicking the physiological complexity. These models also incorporate plasma lipid constituents and potential endogenous TLR-ligands, which may further influence immune activation. [44]. To compare response patterns in T1DM versus healthy controls, we tested three distinct classes of stimuli: clinically relevant alginate microspheres, bioparticles of heat-killed bacterial and fungal species, and selective immune agonists, including TLR ligands. While clinically relevant alginate microspheres are proposed as materials for cell-encapsulation therapy, in this study we tested the materials in the absence of cells to specifically assess how the host immune system responds to the material itself. The alginate microspheres used in this study have been previously characterised in detail [18,36,39]. In the present work, we included the following types: **1.** Alginate microbeads (A) containing High-G alginate (68 % G) previously subjected to transplantation in humans [15,34], representing the pro-fibrotic clinically relevant materials; **2.** Alginate microbeads containing Int-G alginate (47 % G content), reducing fibrosis in mice [36], whereas not yet tested in clinical settings; **3 & 4.** Sulfated alginate (SA) microbeads in two slightly different compositions (SA/A 10/90 and 20/80), both of which show fibrosis reducing potentials in mice [36,39], not yet been involved in clinical transplantation. **5.** Alginate microbeads coated with poly-L-lysine (AP) used as reference, known to induce inflammation [41], as well as fibrosis in mice [45,46]. Considering the altered immune and metabolic landscape in T1DM, we included baseline comparisons of the lipoproteins and metabolic profiles in T1DM and healthy controls.

To the best of our knowledge, this is the first study comparing the whole blood immune reactions to alginate microbeads in individuals with T1DM versus healthy controls, alongside baseline metabolic screening and immune functional testing. This integrated and comprehensive approach, with a larger cohort of blood donors than previously used, allows clarification of how subtle systemic immune and molecular dysregulations in T1DM influence host reactions to the biomaterials, and hence their biocompatibility in the relevant setting.

## 2 Materials and methods

### 2.1 Study participants

This study included totally nineteen participants with long-term type 1 diabetes mellitus (T1DM) and 27 healthy controls. For comparative analyses between T1DM and healthy controls, participators were matched for sex, age and BMI. Three T1DM participants had two healthy control matches. Five healthy controls were excluded from the comparative analyses, due to insufficient matching, which resulted from the withdrawal of five T1DM participants before experiments began. Among the T1DM participants, fifteen were recruited from St. Olav's Hospital, Department of Endocrinology, and four through advertisements in local channels at St. Olav's University Hospital and NTNU. Healthy controls were recruited exclusively through local advertisement at these institutions. Patients were between 18 and 65 years old upon recruitment time-point. Exclusion criteria included pregnancy, breastfeeding, drug or alcohol abuse within the past two years, a history of psychological disorders, and disorders affecting the gastrointestinal system, kidneys, lungs, or malignancies.

Matching criteria for BMI and age were as follows: BMI <25 ± 1.5 kg/m^2^, BMI 25–30 ± 2 kg/m^2^, BMI >30 ± 3 kg/m^2^; Age <35 ± 5 years; Age >35 ± 10 years. Participants were required to be free from colds and NSAID use for 14 days before blood collection. Blood was collected during a study period of eight weeks, with most participants within one month. On the study days, participants arrived in a fasting state (8–10 h) in the morning. Upon arrival, their weight was measured, and information regarding any inflammatory illnesses and intake of NSAIDs use within the past 14 days were recorded.

Among the participants, four controls and two T1DM individuals had used NSAIDs at a single time point (200–400 mg), and one T1DM participant had received a methylprednisolone injection within the previous 14 days. One T1DM and one control did not adhere to the fasting requirement. The clinical characteristics of the participants are summarised in Table 1.

**Table 1:** **Clinical characteristics of the participants.** Values of matched participants are given as geometric mean ± geometric SD. Normal range values for the given parameters are: HbA1c 28–37 mmol/mol, PS-Cholesterol 18–30 years: 2.9–6.1 mmol/L, 30–49 years: 3.3–6.9 mmol/L, ≥50 years: 3.9–7.8 mmol/L, PS-LDL 1.5–5.0 mmol/L, PS-HDL >1.0–2.1 mmol/L, PS-Triglycerides 0.45–2.6 mmol/L ∗Clinical details of T1DM participants (n = 18) were obtained from the latest medical records (Data of a T1DM female participant is missing).

	T1DM (n = 19/18∗)	Ctr (n = 22)	P-Value
Sex (M/F)	13/6	14/8	–
Age (years)	37.70 ± 1.43	39.48 ± 1.36	0.76, ns
BMI (kg/m^2^)	25.61 ± 1.11	25.21 ± 1.11	0.64, ns
HbA1c (mmol/mol)	51.51 ± 1.18∗	–	–
PS-Cholesterol (mmol/L)	4.60 ± 1.21∗	–	–
PS-LDL (mmol/L)	2.84 ± 1.33∗	–	–
PS-HDL (mmol/L)	1.57 ± 1.17∗	–	–
PS-Triglycerides (mmol/L)	0.83 ± 1.47∗	–	–

### 2.2 Blood withdrawal and sample collection

Blood samples from each participant were collected in anticoagulated vacutainers containing either lepirudin (50 μg/mL), heparin (Greiner Bio-one, 456028), or EDTA (Greiner Bio-one, 454209). Human whole-blood experiments based on lepirudin-anticoagulated blood began exactly 20 min after collection (section 2.8.1), while heparin-anticoagulated blood was used within 60 min (section 2.8.2). Plasma separation from EDTA-blood samples commenced within 10 min of collection, after which the plasma samples were stored at −80 ^°^C prior to further analysis (NMR and baseline cytokines).

### 2.3 Alginate polysaccharides

Ultrapure (UP) alginates were acquired from Novamatix (Sandvika, Norway), including low-viscosity high G (LVG; 68 % G, 237 kDa) and low-viscosity high M (LVM; 47 % G, 235 kDa). Medium-viscosity high G (MVG) alginate (66 % G, 233 kDa) was sulfated as described by Arlov et al. [38]. The resulting sulfated alginate had a degree of sulfation (DS) of 0.83 and an expected reduction in average molecular weight (M_w_) to 162 kDa, attributed to hydrolysis caused by chlorosulfonic acid and elevated temperature [47]. ^1^H NMR was employed to assess the G content of alginates using an established protocol by Grasdalen et al. [48,49]. SEC MALLS was used to determine M_w_ [50].

### 2.4 Preparation of microspheres

Alginate microspheres were formulated and gelled according to previously described methods shown to produce stable microspheres under physiological conditions [36,51]. Sterile alginate solutions were prepared at a final concentration of 1.8 % (w/v) in 0.3 M mannitol (Sigma-Aldrich, St. Louis, MO, USA) and 10 mM HEPES (Corning Inc., Corning, NY, USA) at pH 7.2–7.4. For sulfated alginate microspheres, sulfated MVG was mixed with LVG at ratios of 10/90 and 20/80, with final concentrations (w/v) of 0.18/1.62 % and 0.36/1.44 %, respectively. Based on their alginate and sulfated alginate content, the final microspheres were designated High G (LVG), Int G (LVM), SA/A 10/90 (sulfated MVG/LVG ratio 10:90) and SA/A 20/80 (sulfated MVG/LVG ratio 20:80). Microspheres were produced using an in-house built electrostatic droplet generator system (NTNU, Trondheim) equipped with a 0.35 mm nozzle and a syringe pump (Cole-Parmer Instrument Company LLC, Vernon Hills, IL, USA) set to flow rate of 10 mL/h at 6 kV, dripping into a gelation solution of 50 mM CaCl_2_ (Sigma-Aldrich), 1 mM BaCl_2_ (Merck KGaA, Darmstadt, Germany), 0.15 mM mannitol and 10 mM HEPES at pH 7.3–7.4 for 5 min. The microspheres were then removed from the gelling solution and rinsed in a physiologically compatible washing solution containing 0.9 % NaCl (VWR International), 2 mM CaCl_2_ and 10 mM HEPES at pH 7.2–7.4. All gelling and washing solutions were prepared using sterile, hypotonic, nonpyrogenic water (B. Braun Melsungen AG, Germany). Microspheres were further aliquoted (0.5 mL) in vials (Cat 366656, 1,8 mL: Nunc, Roskilde, Denmark) and stored at 4 ^°^C. AP microspheres were prepared by dripping High G alginate in 50 mM CaCl_2_ for gelation, followed by incubation in 0.1 % poly-L-lysine (PLL) (Mw = 15–30 kDa, Sigma-Aldrich) dissolved in 0.9 % NaCl for 10 min to acquire an outer PLL layer. AP microcapsules were washed with 0.9 % NaCl before and after PLL treatment. The diameters of the microspheres ranged from 416 ± 19 to 524 ± 25 μm. Details of the microspheres are summarised in Table 2.

**Table 2:** Overview of the alginate, gelling- and polycations, morphology and diameters (μm) with standard deviations (SD) for the five types of microspheres investigated.

Microsphere type	High G	Int G	SA/A 10/90	SA/A 20/80	AP
Alginate/G content (%)	LVG/68	LVM/47	LVG/68	LVG/68	LVG/68
Ratio SA^a^/A	0/100	0/100	10/90	20/80	0/100
Gelling ions (Ba^2+^/Ca^2+^ in mM)	1/50	1/50	1/50	1/50	0/50
Poly-L-Lysine (0.1 %)	**-**	**-**	**-**	**-**	**+**
Morphology^b^					
Diameter ± SD (μm)	416 ± 19	524 ± 35	451 ± 37	493 ± 39	503 ± 28

a Sulfation degree (DS) was 0.83 on the backbone of MVG alginate (66 % G).

b Scale bare are 500 μm.

### 2.5 Bioparticles

Heat-killed bacterial and fungal substances were obtained from Invitrogen (San Diego, USA), including HKEB (Heat-killed *Escherichia coli* 0111: B4), HKSP (Heat-killed *Streptococcus pneumoniae*), HKMT (Heat-killed *Mycobacterium tuberculosis*) and HKCA (Heat-killed *Candida albicans*). Standard stock solutions were prepared and stored according to the manufacturer's instructions. Optimal working concentrations were selected for each stimulant based on experimental requirements. For stimulation of blood cells, HKEB, HKSP and HKCA were used at a concentration of 10^8^ cells/mL, while HKMT was used at 100 μg/mL.

### 2.6 Selective immunostimulatory ligands

For the immune functional assay (IFA) blood model (section 2.8.2) immune agonists from InvivoGen were used at the following final concentrations: TLR2 ligand (FSL-1, 100 ng/mL), TLR3 ligand (poly I:C, 20 μg/mL), TLR4 ligand (LPS O111:B4, 1 ng/mL), TLR7 ligand (Gardiquimod, 1 μg/mL), TLR8 ligand (TL8-506, 300 ng/mL), dectin ligand (whole glucan particle (WGP®, 40 μg/mL), and STING ligand (2′3′- cGAMP, 13 μg/mL). The polyclonal T cell stimulant (human CytoStim, 2 μL/mL) was from Miltenyi Biotec and was potentiated by the addition of a co-stimulatory anti-CD28 mAb (InVivoMAb, BioXCell, 300 ng/mL).

### 2.7 Proton nuclear magnetic resonance (^1^H NMR) analysis

Metabolomic profiles of each participant were analysed using ^1^H NMR, following the standard operating procedures of Bruker *in vitro* Diagnostics Methods (B.I.Methods^TM^) [52]. EDTA anticoagulated plasma samples (350 μL) were thawed and mixed with an equal amount of sodium phosphate buffer (0.075 M, pH 7.4, 20 % D_2_O in H_2_O, 6 mM NaN_3_, 4.6 mM 3-(trimethylsilyl)-2,2,3,3-tetradeuteropropanoic acid) (TSP-d4). Mixtures were transferred to NMR tubes (5 mm diameter, 40 mm fill height). NMR spectra were recorded using a Bruker Avance Neo 600 MHz spectrometer equipped with a 5 mm BBI probe (Bruker Biospin GmbH, Rheinstetten, Germany). Data acquisition and sample handling were performed by IconNMR (Topspin 4.1.3, Bruker Biospin GmbH) and SampleJet autosampler (Bruker Biospin GmbH), respectively. A sequence of experiments consisting of one-dimensional nuclear Overhauser effect (1D-NOESY) with water presaturation, a diffusion and relaxation edited (DIRE) experiment (1D-PGPE), and 2D-JRES were recorded at 310 K. The 1D spectral data were collected using 96k data points, 30-ppm spectral width, and 32 scans. Free induction decays were zero-filled to 128 k points, 0.3 Hz line broadened, and Fourier transformed. Spectra were automatically zero-order phase corrected.

Bruker PhenoRisk PACS (Post-Acute COVID Syndrome) RuO 1.0.0 analysis panel [53] was used for quantification of 16 risk markers relevant to diabetes, inflammation, cardio and kidney disorders (glucose, creatinine, glycoproteins (Glyc, GlycA, GlycB), supramolecular phospholipid composite (SPC), total triglycerides, total cholesterol, cholesterol and phospholipid contents of LDL/HDL particles, and apolipoproteins (Apo-A1, Apo-B100). Other baseline compounds like amino acids (2-aminobutyric acid, alanine, asparagine, creatine, creatinine, glutamic acid, glutamine, glycine, histidine, isoleucine, leucine, lysine, methionine, N,N-dimethylglycine, ornithine, phenylalanine, proline, sarcosine, threonine, tyrosine, and valine), carboxylic acids (2-hydroxybutyric acid, acetic acid, citric acid, formic acid, lactic acid, succinic acid) and keto acids (2-oxoglutaric acid, 3-hydroxybutyric acid, acetoacetic acid, acetone and pyruvic acid) were assessed by Bruker IVDr Quantification in Plasma/Serum (B.I.Quant-PS^TM^2.0) [54].

Further, Bruker IVDr Lipoprotein Subclass Analysis (B.I.LISA^TM^) [55] was employed to quantify the plasma concentration of major lipoproteins; VLDL (0.950–1.006 kg/L), IDL (1.006–1.019 kg/L), LDL (1.019–1.063 kg/L) and HDL (1.063–1.210 kg/L), as well as the lipoprotein subclasses; VLDL-1 to 5, LDL-1 to −6, and HDL-1 to −4 [47] and these subclasses were classified according to ascending density ranges. Additionally, B.I.LISA analysis quantified all the sub-components: triglycerides (TG), cholesterol (CH), free cholesterol (FC), phospholipids (PL) and apolipoproteins (Apo-B100, Apo-A1, Apo-A2) of lipoproteins.

### 2.8 Human whole blood models

#### 2.8.1 Lepirudin-based model

The lepirudin-based human whole-blood model originally developed by Mollnes et al. (2002) [56], and later adapted for microsphere evaluations [40,57], was used to investigate acute phase responses, including coagulation, complement activation and early cytokine induction. Microspheres (50 μL) were aliquoted in low-activating polypropylene vials (Cat 366656, 1,8 mL: Nunc, Roskilde, Denmark) containing 50 μL 0.9 % NaCl (B. Braun, Melsungen, Germany), and then added 100 μL PBS with CaCl_2_ (133 mg/L) and MgCl_2_ (100 mg/L). Glass tubes (Cat:367614, BD Vacutainer) and PLL microcapsules were included as positive controls for coagulation activation and complement activation, respectively. Microspheres and controls were added to 500 μL whole blood anticoagulated with lepirudin (50 μg/mL) and incubated at 37 ^°^C for 60 min or 240 min under continuous rotation. Complement and coagulation activation were stopped by adding EDTA (Sigma-Aldrich) to a final concentration of 10 mM. Plasma samples from each condition were collected and aliquoted after centrifugation at 3000×*g* for 15 min. Samples were stored at −20 ^°^C for further analysis. Samples from nineteen T1DM participants and matched 22 controls (double match for three diabetics) were included in this analysis.

#### 2.8.2 Heparin-based dilution model

An IFA with whole blood (TruCulture) was previously developed for immune monitoring at clinical sites [58], which minimises sample handling to reduce experimental bias. Inspired by this approach, we established a similar in-house IFA for our study. Peripheral venous blood from T1DM participants and matched healthy controls was collected in 6 mL VACUETTE®NH Sodium Heparin tubes (Greiner Bio-One). Within 60 min after blood collection, aliquots of 250 μL were added to polypropylene stimulation tubes (Cluster tubes, Corning) containing 500 μL RPMI cell culture medium, 10 U/mL sodium heparin, and various immune agonists. To minimise technical variation, immune activators were pre-diluted in RPMI in large batches, aliquoted into the stimulation tubes, and stored at −20 ^°^C until use. Once thawed at RT, fresh blood was added at a 1:3 dilution and gently mixed by pipetting 6 times. Samples were incubated at 37 ^°^C and 5 % CO_2_ under static conditions for 18 or 72 h. During incubation, blood cells sedimented via gravity, and supernatants were collected and stored at −20 ^°^C. The stimulant concentration and incubation time were optimised in pilot experiments to elicit robust cytokine responses while avoiding saturation effects. For 18 h of stimulation, we included the non-stimulated baseline (RPMI alone) and RPMI with selective agonists for TLRs, Dectin-1 and STING (as detailed in section 2.6). T cell responses were assessed after 72 h, under non-stimulated conditions (RPMI alone) and with a synthetic superantigen (CytoStim) that cross-binds TCR and MCH in combination with co-stimulating anti-CD28 mAb. Samples from nineteen T1DM participants and controls were analysed.

### 2.9 Fluidic phase TCC quantification

Complement activation was assessed by quantifying soluble terminal complement complex (TCC) using a sandwich ELISA kit (Hycult Biotech, HK328) according to the manufacturer's instructions. TCC levels were measured after 60 min for bacterial substances and 240 min for other conditions. To correct for plate-to-plate variations, samples were consistently arranged in paired dual combinations (T0 & Saline, Int G & SA/A 10/90, High G & SA/A 20/80, HKEB & HKCA, HKSP & HKMT). Each diabetic donor sample was processed alongside an age- and BMI-matched healthy control sample.

### 2.10 Coagulation activation

Prothrombin fragment F 1 + 2 (PTF1.2) levels were measured to evaluate coagulation using the Enzygnost F 1 + 2 monoclonal ELISA kit (Siemens Healthcare Diagnostics, Marburg, Germany) following the manufacturer's protocol. Sample dilutions ranged from 10 × to 1000 × , depending on the stimuli. Plate-to-plate variations were eliminated as described in section 2.9.

### 2.11 Cytokine profiling

Multiplex cytokine assays (Bio-Plex Human cytokine 27-Plex Panel or selected cytokines, Bio-Rad Laboratories, Hercules, CA) were used to analyse cytokine levels in plasma supernatants. For microspheres the 27-Plex panel was used; IFN-γ (IFNG), TNF, interleukins; IL-1β, IL-1ra (IL1RN), IL-2, IL-4, IL-5, IL-6, IL-7, IL-8 (CXCL8), IL-9, IL-10, IL-12 (IL-12p70), IL-13, IL-15, IL-17, chemokines; Eotaxin (CCL11), IP10 (CXCL10), MCP-1 (CCL2), MIP-1α (CCL3), MIP-1β (CCL4), RANTES (CCL5), growth factors: b-FGF (FGF2), G-CSF (CSF3), GM-CSF (CSF2), PDGF-BB (PDGFB), VEGF. For bioparticles and TLR-ligands the panel included; IFN-γ (IFNG), TNF, IL-1β, IL-2, IL-4, IL-6, IL-8 (CXCL8), IL-12 (IL-12p70), Eotaxin (CCL11), IP10 (CXCL10), and PDGF-BB (PDGFB). For the T-cell stimuli the panel included; IFN-γ (IFNG), TNF, IL-1β, IL-2, IL-6, IL-12 (IL-12p70), IL-17, MCP-1 (CCL2), Eotaxin (CCL11), IP10 (CXCL10). Multiplex assays were performed with half the volume of samples. To minimise plate-to-plate variations, samples were processed using randomised pairing. The first run included saline, Int G, High G, SA/A 10/90, and SA/A 20/80 samples of each paired match placed in the same plate (subject-based sorting). In the second run, samples from all participants with the same stimuli were run together (stimulus-based sorting). No significant differences were detected between the two runs, confirming data consistency.

### 2.12 Statistical analysis

Statistical analyses were performed using GraphPad Prism (v10.3.0). Data distributions were assessed using normality and lognormality tests. Log-transformation was applied to datasets exhibiting lognormal distribution to better approximate normal distribution. To compare multiple variables between two groups, we performed multiple t-tests, assuming similar variance per variable in the two groups. False Discovery Rate (FDR) correction was applied using the two-stage step-up method of Benjamini, Krieger and Yekutieli (desired FDR: Q = 5 %). Results were visualised as volcano plots, and selected analytes were additionally shown as dot plots with individual q-values and uncorrected p-values from the t-tests.

To evaluate differences in *ex vivo* functional blood response between T1DM and control groups, we performed Principal Component Analysis (PCA) on the subject level. PCA settings included data scaling and PC selection based on parallel analysis, typically retaining 4–5 PCs for further investigation. Group differences along selected PCs were tested using 2-way RM ANOVA with Geisser-Greenhouse correction (α = 0.05), followed by Tukey's multiple comparisons test with adjusted p-values (α = 0.05) for pairwise comparisons. We also compared the responses induced by the individual treatments in the T1DM and control groups using unpaired multiple t-tests (Q = 5 %) of the original data, as graphed by volcano plots and dot/bar plots.

To compare the *ex vivo* blood response profiles induced by different microspheres, we merged the data from the T1DM and control groups, including the five non-matched controls, and performed PCA at the sample level. Response variance per treatment was visualised with bar plots of PC scores and PC loadings, and a selected 2D PC scores plot. Response profiles were directly compared using multiple unpaired t-tests of the original data (volcano plots), and selected variables plotted as bar/dot plots with 1-way RM ANOVA (α = 0.05) and Tukey's multiple comparison test (α = 0.05).

### 2.13 Ethics

This study was approved by the Regional Committee for Medical and Health Research (REK) under application number 44255, in compliance with the Norwegian Health Research Act, §10.

## 3 Results

### 3.1 ^1^H NMR analysis reveals distinct lipidomic and metabolic plasma profiles in T1DM

Lipidomic and metabolic plasma profiles of each participant were analysed using ^1^H NMR spectroscopy. We first examined 16 analytes using the Bruker IVDr Post-Acute COVID-19 Syndrome (B.I.PACS) system, which includes biomarkers relevant to diabetes, cardiovascular disease (CVD), kidney disease (KD), and inflammation. The volcano plot (Fig. 1A) shows that the plasma concentrations of all biomarkers, except for creatinine and total cholesterol, differed significantly (q < 0.05) between the groups. The primary glycaemic marker, random blood glucose (RBG), showed the largest increase in the T1DM group (1.2-fold, Fig. 1A–B). Intriguingly, the lipidomic profiles demonstrated significantly elevated HDL and reduced LDL levels in T1DMs compared to healthy controls, including both the phospholipid and cholesterol components of HDL and LDL particles. The LDL/HDL cholesterol ratio displayed the largest reduction of all the variables in the T1DM group (1.4-fold, Fig. 1A–B), which aligned with a decreased ApoB100/A1 ratio, where ApoB100 and A1 are the apolipoprotein fractions of non-HDL (LDL, IDL and VLDL) and HDL particles, respectively (Fig. 1A–B). Glycoprotein signals (GlycA, GlycB, Glyc), which reflect the glycosylation patterns of circulating acute-phase inflammatory proteins, especially fibrinogen, α1-antichymotrypsin, haptoglobin-1, α1-antitrypsin, complement C3, and α1-acid glycoprotein [[59], [60], [61]], were significantly reduced in T1DM participants. SPC (supramolecular phospholipid composite), an NMR signature reflecting phospholipids in lipoproteins, choline head groups in α1-acid glycoprotein, and N-acetyl moieties linked to GlycA and GlycB signals [61], was elevated in T1DM (1.2-fold). Consequently, the Glyc/SPC ratio was markedly lower in T1DM individuals (Fig. 1A–B).

**Fig. 1:**
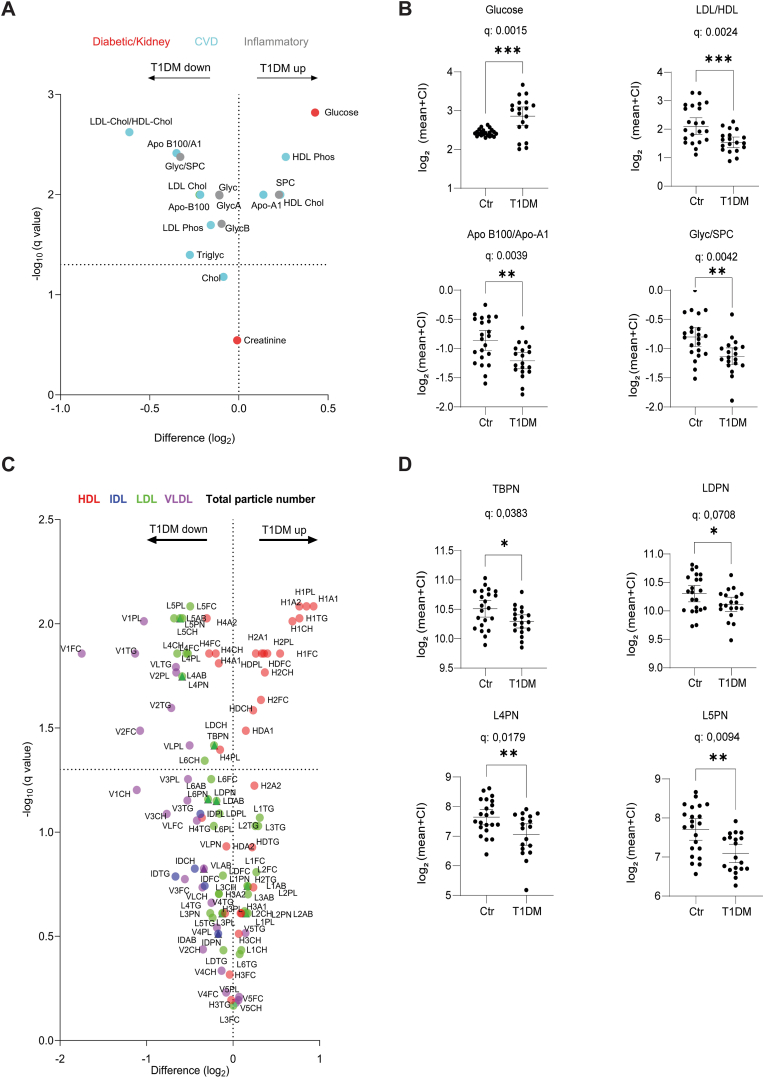
**Metabolic and lipidemic landscape of T1DM and healthy controls. (A)** Volcano plot of analytes relevant to CVD/KD and inflammation in subjects with T1DM (n = 19) and healthy controls (n = 22). **(B)** Scatter plot of individual values of Glucose (Random blood glucose), LDL/HDL–HDL-cholesterol ratio, ApoB100/ApoA1 ratio, and the ratio between composite signals of Glyc and SPC. **(C)** Detailed comparison of major lipoprotein classes (HDL, LDL, VLDL and IDL) and subclasses in the groups, with lipoprotein particle numbers (PN) and molecular composition. **(D)** Scatter plots of total particle numbers for ApoB100-carrying particles (TBPN and LDLPN), as well as LDL-4 and LDL-5 subclasses (L4PN and L5PN).

We further assessed lipoprotein subclasses using Bruker IVDr Lipoprotein Subclass Analysis (B.I.LISA^TM^) NMR. In addition to a quantifying cholesterol and phospholipid concentrations of the four main lipoprotein classes (HDL, IDL, LDL, and VLDL), this method quantifies each component of all the lipoprotein subclasses (HDL-1 to 4, LDL-1 to 6, and VLDL-1 to 5), as well as the levels of triglycerides (TG), cholesterol (CH), free cholesterol (FC), phospholipids (PL), and apolipoproteins (ApoA1 and ApoA2 in HDL, Apo-B100 (AB) in IDL, LDL and VLDL) (Fig. 1C). Stacked bar charts further illustrate the proportional composition of these components within the lipoprotein particles (Fig. S1A).

Among the four HDL subclasses, the HDL-1 and 2 were significantly enriched in T1DM plasma (Fig. 1C), including all the examined HDL-1 constituents (H1A1, H1A2, H1PL, H1CH, H1TG, and H1FC) and most HDL-2 constituents (H2PL, H2A1, H2CH, and H2FC). In contrast, HDL-4 components (H4TG, H4CH, H4FC, H4PL, H4A1, H4A2) were slightly but significantly reduced (Fig. 1C). Among the five VLDL sub-classes, VLDL-1 and 2 were markedly lower in T1DM, as were LDL-4 and LDL-5-associated components (Fig. 1C).

Non-HDL lipoprotein concentrations were estimated based on their corresponding ApoB100 levels, given the 1:1 stoichiometry per particle. The total amount of Apo-B100 containing particles, calculated as total particle number (TBPN), was reduced in T1DM compared to healthy controls (Fig. 1D). The numbers of LDL, IDL, and VLDL particles (LDPN, IDPN and VLPN, respectively) and LDL subclass particles (L1PN, L2PN, L3PN, L4PN, L5PN and L6PN) revealed that L4PN and L5PN were significantly lower in T1DMs compared to healthy controls (Fig. 1D). While an unpaired *t*-test indicated a significant reduction in total LDL particles (LDPN), this difference was not significant after FDR correction (q = 0.0708, Fig. 1C–D).

Normalisation of non-HDL lipoprotein components to their respective particle numbers revealed a reduction of triglycerides in the VLDL fraction in T1DM. However, no other significant differences were observed in the lipoprotein composition between groups (Fig. S1B). This suggests that alterations in the lipoprotein profiles of T1DM individuals mainly stem from changes in lipoprotein subclass particle numbers rather than the molecular composition of the lipoproteins. In addition, no significant differences were detected in other metabolites, including amino acids, carboxylic acids, keto acids and cytokines (Fig. S1C–D)

### 3.2 Functional assays reveal similar acute-phase responses in whole blood from T1DM participants and healthy controls

The comparison of whole blood responses between T1DM and controls included three sets of immune stimuli: (i) microspheres (Int G, High G, SA/A 10/90, SA/A 20/80, and AP), (ii) bacterial/fungal bioparticles (HKEB, HKSP, HKMT and HKCA) and (iii) selective agonists targeting distinct immune sensors (various TLR ligands, a STING ligand, and a polyclonal T cell activator). To analyse these comprehensive data sets, multivariate principal component analysis (PCA) was used. For the microsphere-treated samples, the response variables include 27 cytokines, TCC and PTF1.2 in 41 subjects (19 T1DM and 22 controls), thus comprising 1189 variables. PCA was performed on the subject level (wide format) to assess clustering between the T1DM and control groups. The number of variables was reduced to five principal components (PCs), capturing 63.7 % of total variance (Fig. 2AB). A 2D score plot of PC1 vs. PC2, representing the largest variance, showed no distinct separation between T1DM and controls, indicating similar responses to the microspheres (Fig. 2A). Statistical testing of all five PCs supported this, with no significant differences between groups (2-way ANOVA, overall p > 0.9999) (Fig. 2B). This was further corroborated by univariate statistics, using multiple unpaired t-tests with FDR control (Q = 5 %), as visualised in volcano plots (Fig. 2C). The only significant difference was a slightly lower TCC concentration in saline-treated T1DM blood compared to controls (q < 0.05) (Fig. 2C). Bar plots further depict individual cytokine, PTF1.2 and TCC highlighting their overall similarity between groups (Fig. S2).

**Fig. 2:**
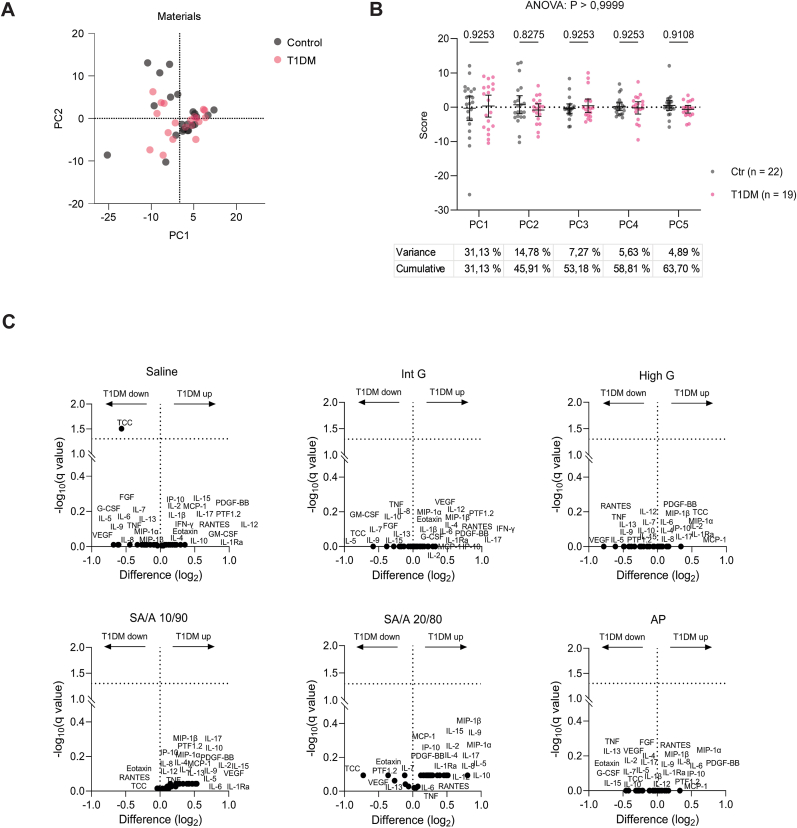
**Immune functional testing of distinct microspheres with human whole blood from T1DM and healthy controls.** Multiple immune response parameters (n = 29) in blood from T1DM patients (n = 19) and controls (n = 22) after ex-vivo treatment with saline and distinct microspheres, as analysed by Principal Component Analysis (PCA) to compare the study groups. **(A)** Response to five microspheres and saline control visualised by PC1 vs PC2 plot, **(B)** Dot plot of individual PC (PC1 to PC5) score values with the associated overall ANOVA and pairwise post-test p-values, and the variance the PCs cover. **(C)** Volcano plots of each stimulus comparing the induction of each variable in the two groups.

The response variables examined after treatment with bioparticles (HEKB, HKMT, HKSP, and HKCA) were 11 cytokines (IFN-γ, IL-1β, IL-6, TNF, IP-10, IL-8, IL-2, IL-4, IL-12, Eotaxin, PDGF-BB), PTF1.2, and TCC. The dataset, comprising 520 variables in 40 subjects (18 T1DM and 22 controls), was reduced to five PCs, retaining 69.58 % of total variance. The set of one T1DM participant was removed by statistical software due to a missing value in IP-10 cytokine analysis. Again, PCA revealed no clear separation between groups (overall p = 0.9998) (Fig. 3A and B). This was also consistent with univariate analysis of the original data, as visualised by Volcano plots (Fig. 3C) and bar plots (Fig. S3) (q > 0.05). Here, we only observed a non-significant trend of reduced TCC and PTF1.2 activation by heat-killed *Mycobacterium tuberculosis* (HKMT) in T1DM.

**Fig. 3:**
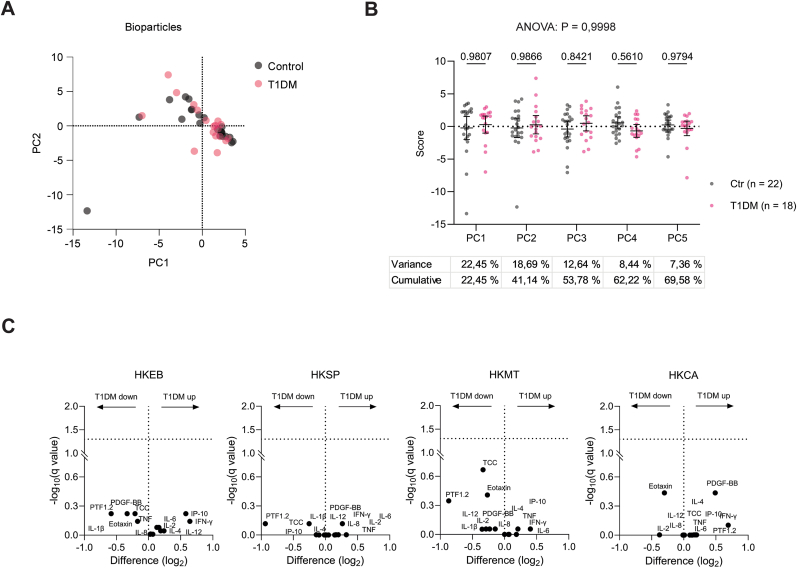
**Immune functional testing using bioparticles (HKEB, HKSP, HKMT and HKCA) and human whole blood from T1DM and healthy controls.** Multiple immune response parameters (n = 13) in blood from T1DM patients (n = 18∗) and controls (n = 22) after ex-vivo treatment with distinct bioparticles were analysed by Principal Component Analysis (PCA) to compare the study groups. **(A)** Response to four bioparticles visualised as a PC1 vs PC2 plot. **(B)** Dot plot and statistical testing of individual PC (PC1 to PC5) scores**,** showing the overall ANOVA and pairwise post-test p-values, which do not reveal any significant differences between the groups. The variance of the original data that the PCs cover is shown below. **(C)** Volcano plots comparing the induction of response variables in the groups by distinct bioparticles, revealing no significant differences. ∗Values from one donor are omitted due to statistical software requirements explained under section 3.2.

The response variables examined after treatment with selective immune-receptor ligands (TLR2L, TLR3L, TLR4L, TLR7L, TLR8L, Dectin-L, STING-L) were 11 cytokines for the ligands and 10 cytokines for the T cell stimuli, yielding 98 combined variables in a wide-format PCA setup (Fig. 4A–B). The five selected PCs covered 56.44 % of the total. Again, PCA indicated no significant differences between T1DM and control responses (overall p > 0.9999), which was further supported by unpaired multiple t-tests (Fig. 4C) (Fig. S4).

**Fig. 4:**
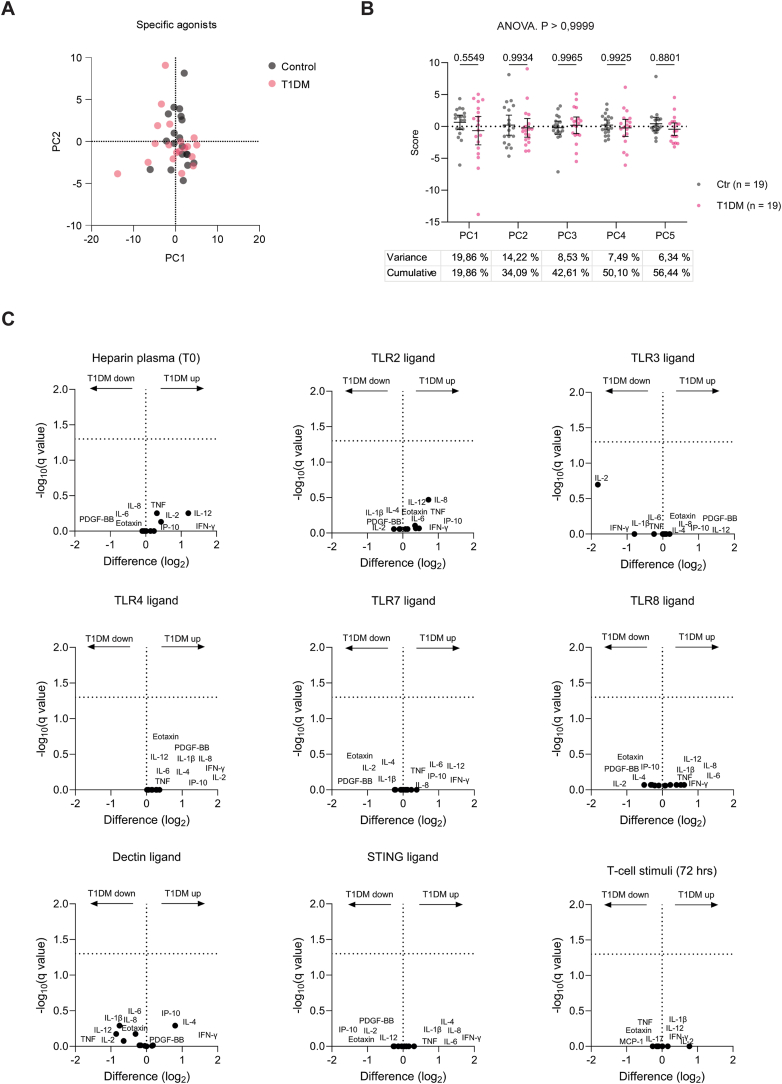
**Immune functional testing using distinct TLR ligands (TLR2, TLR3, TLR4, TLR7, TLR8) ligands for Dectin and STING, and a T cell stimulus with human whole blood from T1DM and healthy controls.** Multiple immune response parameters (n = 11) in blood from T1DM patients (n = 19) and controls (n = 19) after ex-vivo treatment with distinct TLR ligands were analysed by Principal Component Analysis (PCA) to compare the study groups. **(A)** Response to TLR ligands visualised as a PC1 vs PC2 plot. **(B)** Dot plot and statistical testing of individual PC (PC1 to PC5) scores, showing the overall ANOVA and pairwise post-test p-values, which do not reveal any significant differences between the groups. The variance of the original data that the PCs cover is shown below. **(C)** Volcano plots comparing the induction of response variables in the groups by distinct TLR ligands, revealing no significant differences.

In essence, *ex vivo* whole blood responses to immune stimuli were highly similar between the T1DM and control groups. No significant differences were observed, except for a slightly lower TCC level in saline-treated T1DM blood.

### 3.3 Inflammatory signature of alginates microspheres with clinical relevance

Given the comparable immune responses between T1DM and controls, data from the groups were merged for further analysis of microsphere-induced responses, incorporating 5 additional unmatched controls. This dataset included lepirudin-anticoagulated blood samples from 46 subjects (19 T1DM and 27 controls) treated *ex vivo* with four clinically relevant microspheres (Int G, High G, SA/A 10/90, SA/A 20/80) or controls (saline, AP microspheres). We quantified 29 response variables (TCC, PTF1.2 and 27 cytokines) and performed PCA on the sample level (long format setup) to assess sample clustering. The four major PCs (74.79 % cumulative variance) revealed distinct immune response patterns for poly-L-lysine containing microspheres (AP), unmodified alginate microspheres (Int G, High G), and sulfated alginate microspheres (SA/A 10/90, SA/A 20/80) (Fig. S5A). A 2D score plot of PC1 vs. PC3 clearly illustrates the overall distinction of these three microsphere groups, while the two sulfated alginate microspheres, as well as the two non-modified alginate microspheres, were pairwise clustered (Fig. 5A). Notably, PC1 captured the highly distinct immune response to the AP control microsphere (Fig. 5A and S5A), as all variables except Eotaxin exhibited a positive loading. This underscores the high pro-inflammatory potential of these microspheres (Fig. S5A).

**Fig. 5:**
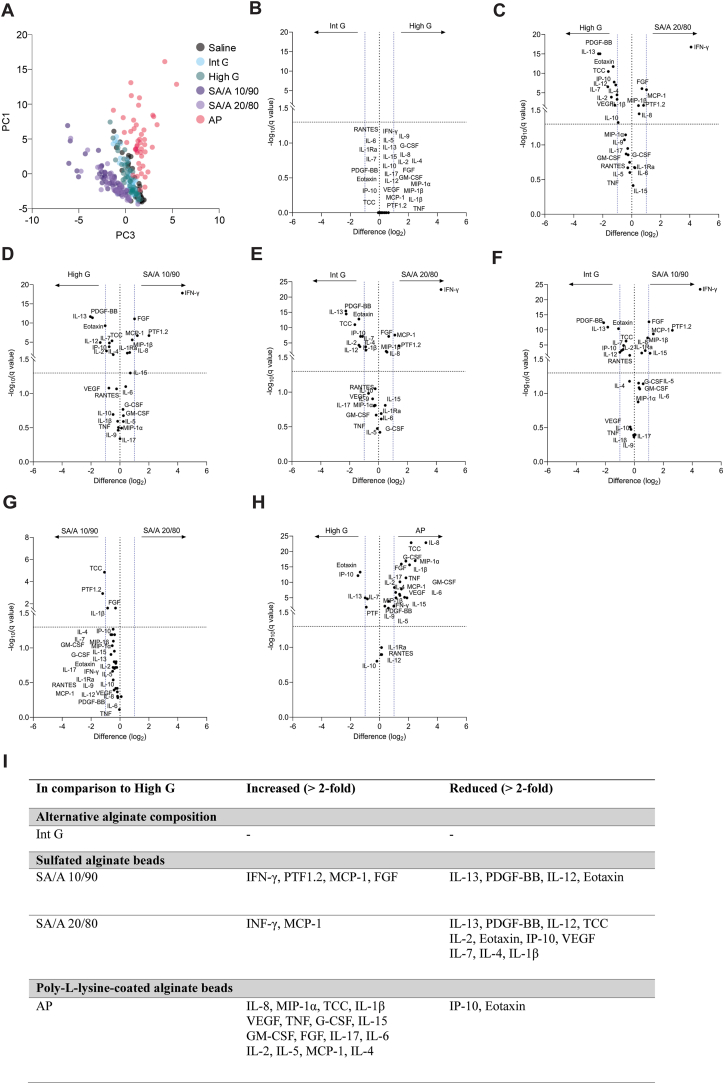
**Immunesignaturesinducedbythedifferentalginatemicrospheres**. (A) *PCA 2D plot of 29 response parameters in whole blood from 46 subjects (19 T1DM and 27 controls) with 6 treatment conditions, revealing distinct response signatures of the microsphere types. The volcano plots show a direct comparison of the responses to****(B)****High G versus Int G****(C)****High G versus SA/A 20/80****(D)****High G versus SA/A 10/90****(E)****Int G versus SA/A 20/80****(F)****Int G versus SA/A 10/90****(G)****SA/A 10/90 versus SA/A 20/80,****(H)****High G versus AP****(I)****Summarised parameters giving two-fold increase/decrease changes compared to High G alginate microbeads. Stippled horizontal lines indicate the adjusted significance threshold (*q > 0.05) *and stippled vertical lines mark the 2-fold change boundaries.*

The distinctions between sulfated and non-sulfated are reflected by PC2, PC3, and PC4 dimensions (Fig. S5A). For a more direct and focused comparison, we performed multiple paired t-tests of response variables with correction for multiple testing (Q = 5 %), as visualised by volcano plots (Fig. 5B–H). Int G and High G did not differ significantly regarding cytokine induction (q > 0.05, Fig. 5B). The comparison of clinically relevant microbeads such as SA/A 20/80 with their baseline material, High G, indicated that sulfation modification significantly elevated 6 of the 29 inflammatory mediators examined (q < 0.05), with a marked increase in the level of IFN-γ. Conversely, SA/A 20/80 microbeads reduced the levels of 12 mediators, including IL-13, PDGF-BB, IL-12, and TCC (Fig. 5C). SA/A 10/90 reduced 8 mediators significantly compared to High G (Fig. 5D). Moreover, as could be expected, sulfated alginate microbeads had similar effects when compared to Int G (Fig. 5E and F). Pairwise comparison of SA/A 20/80 and SA/A 10/90 revealed that the alginate composition with a higher degree of sulfation significantly lowered the levels of TCC, PTF1.2, IL-1β, and FGF (Fig. 5G). The AP microbeads significantly increased the levels of 20 mediators and diminished 5 mediators when compared to High G (Fig. 5H). Most strongly increased by AP were IL-8, MIP-1α, TCC and IL-1β. The effects of the various microspheres relative to the baseline material (High G) on whole blood inflammatory mediators are summarised in a tabular form (Fig. 5I), limited to those that were more than 2-fold changed. The additional comparisons (AP versus Int G and AP versus sulfated alginate) are detailed in Supplementary Fig. S5B–C. The AP versus Int G comparison exhibits overall similar trends to those observed in High G. In comparison to SA, the AP treatment induces an elevation in 21 out of 29 mediators.

To explore individual response variables across treatment conditions, we visualised all markers by dot plots (Fig. S5D). All variables shown exhibited significant treatment-induced changes (One-way-RM ANOVA, overall p < 0.05), and agree with the pairwise testing results in Fig. 5.

## 4 Discussion

In this study, we examined the *ex vivo* immune responses in whole blood from individuals with Type 1 Diabetes Mellitus (T1DM) and healthy controls matched for sex, age, and BMI. We also compared baseline levels of lipoproteins, metabolites and cytokines, revealing significant differences in random plasma glucose and lipoprotein fractions. Specifically, T1DM displayed reduced LDL-4 and LDL-5 particles, lower VLDL triglyceride content, and increased HDL-1 and HDL-2 subclass variables. Despite these metabolic differences, the *ex vivo* immune responses to microspheres, bioparticles and selective immune agonists were similar in T1DM and healthy controls. This suggests that similar inflammatory responses towards foreign objects can be found regardless of the differences in the metabolic and inflammatory state of the T1DM and healthy controls. Further, it shows that the whole blood from healthy donors can serve as a reliable substitute for T1DM patient blood for functional studies of inflammatory responses to biomaterials.

This study encompasses a spectrum of immune initiators, from alginate microspheres to bioparticles and TLR-ligands. The whole-blood model preserves protein cascades and cellular interactions, providing a more physiologically relevant assessment than reductionistic models with purified cells or cell lines. The inflammatory reactions to microspheres are strongly influenced by complement and coagulation cascades [41,42,57], which are almost functionally intact due to the use of a direct thrombin inhibitor [56,57]. Although previous studies reported reduced complement C3 in T1DM [22], potentially lowering complement activation by microspheres, these findings indicate that the TCC levels remain unchanged. This suggests that complement factor redundancy and regulatory complexity compensate for potential baseline differences in C3 levels.

Bacterial and fungal bioparticles can trigger immune pathways, including complement, coagulation and pattern recognition receptors (PRRs) like toll-like receptors (TLRs) [56,62,63]. Given the elevated levels of TLRs (cellular and circulating) in T1DM [30,[64], [65]], increased cytokine induction upon stimulation might have been expected. However, our study found no such differences. Cytokine responses to various TLR ligands were also similar in T1DM and matched controls. This contrasts with a previous study that reported increased IL-1β and TNF production in T1DM, correlating with higher TLR2 and TLR4 expression in monocytes [30]. Further, long-term T1DM patients have been reported with reduced expression of TLR9. However, in new-onset or at-risk patients, TLR9 levels were increased, highlighting the complex immune environment in T1DM patients across disease progression [66,67]. The duration and progression stage of T1DM could likely influence various immune factors, and TLR expression patterns in T1DM are indeed influenced by age, etiology, disease onset and co-morbidities such as ketoacidosis and microvascular complications [64,66,68,65]. T1DM can increase susceptibility to infection with *Mycobacterium tuberculosis*, *Staphylococcus aureus*, and *Candida albicans* [69]. Purified PBMCs from individuals with T1DM showed reduced cytokine production in response to these pathogens [69,70]. Our results, which suggest no significant differences in cytokine responses, may reflect a cohort of well-managed T1DM patients with optimal long-term blood glucose control (HbA1c). Additionally, whole blood represents a functional outcome influenced by multiple factors, rather than the expression of individual factors, which may account for discrepancies with previous research. Whole blood models further avoid biases introduced by leukocyte separation and culture conditions, providing a robust and reliable system for evaluating acute immune responses [71].

Although immune responses showed no significant differences between individuals with T1DM and healthy controls, distinct variations became evident among various clinically relevant microspheres. The multivariate analysis (PCA) clearly distinguished responses induced by alginate and sulfated alginate-containing microspheres. Our previous studies have demonstrated that sulfation of alginate reduces complement activation [36,72], while it modestly increases coagulation [36,39]. However, the statistical support for these effects has been variable. The current study examines a larger cohort of blood donors, providing more robust statistics to differentiate the responses induced by the various biomaterials. Here we confirm that the sulfation of alginate reduces complement and increases coagulation with statistically robust data. Notably, we further show that even minor variations in sulfated alginate amounts can be significant, as higher sulfation levels notably decrease TCC the most, whereas lower sulfation levels significantly increase PTF1.2. Furthermore, due to the larger cohort in the current study, SA/A microbeads are clearly and significantly reducing several of the measured cytokine levels. The cytokines downregulated by more than twofold include IL-13, PDGF-BB, IL-12, Eotaxin (CCL11), IP-10 (CXCL10), VEGF, IL-7, IL-4, and IL-1β, and could thus be among the more relevant in regard to reducing fibrosis. Interestingly, IFN-γ was strongly induced (approx. 16-fold) along with markedly elevated MCP-1 (CCL2; approx. 2-fold). The effect on IFN-γ is consistent with our earlier findings using SA/A 20/80 microbeads, while the pronounced elevation in MCP-1 was not revealed in the former study [39]. IFN-γ is a well-known Th1/NK-cell-derived cytokine that promotes macrophage polarisation toward the M1 phenotype, which is characterised by a shorter lifespan [73,[74], [75]]. MCP-1 is primarily recognised as a monocyte chemoattractant and has also been reported to correlate strongly with IFN-γ [76].

In comparison to high-G and sulfated alginate, the AP microcapsules modulated most of the mediators examined, indicating they have a substantial pro-inflammatory potential, in line with the patterns we previously reported [39,41]. In the current study, we also show that interleukins secreted by T-cells, and growth factors like G-CSF, GM-CSF and FGF are significantly elevated by AP microbeads. On the other hand, IP-10 and Eotaxin are diminished considerably, corroborating our recent observation [39].

Sulfation of alginate increases anionic charge density and promotes interaction with proteins [[77], [78]] enhancing the complement-inert properties of alginate by binding complement inhibitors, thereby reducing TCC induction [[39], [72], [78]]. Importantly, unmodified alginate also has an affinity for complement and coagulation inhibitors, although to a lesser extent [[39], [72], [78]]. Additionally, sulfation increases FXII binding and activation at the surface [[39], [78]] explaining the observed increase in PTF1.2 levels.

The distinct effects of microbeads in the human whole blood model are likely influenced by several factors, including surface charge and bead size. Concerning surface charge, alginate microbeads have been demonstrated to carry a negative surface charge under close-to-physiological ionic strength [79], whereas PLL-coating has been shown to increase the net positive charge [80]. Studies have further demonstrated that PLL only partially complexes with alginate [81,82], suggesting that some positively charged amine groups remain exposed on the surface. We have demonstrated that PLL-coated high-G alginate (AP) exhibits a distinct protein adsorption profile compared to sulfated alginate (SA/A), which represents a more anionic surface [39]. Additionally, AP is associated with activation of the complement system via the alternative pathway, characterised by extensive deposition/activation of complement component C3 [39]. This AP-mediated C3 activation promotes leukocyte adhesion through interaction between the active complement component, C3b/iC3b and the cell surface receptor CR3, accompanied by the release of inflammatory cytokines [41,42]. Conversely, microbeads containing sulfated alginate have been shown to elicit reduced inflammation [[39], [72]]. The pronounced enrichment of complement inhibitors, C1-Inhibitor and factor H, may explain the low complement activation by sulfated alginate microbeads [[39], [78]]. Strategies such as masking amine groups or thinning of the PLL layer in the microcapsules have resulted in diminished immune recognition and reduced fibrosis [79,83].

The influence of alginate bead size on the foreign body response has been examined in rodents and non-human primate models [84], and here the larger alginate beads (1.5 mm diameter) triggered weaker foreign body reactions compared to microbeads (0.5 mm diameter) [84]. In the present study, all the alginate microbeads were produced with a similar size range (0.4–0.5 mm), and we did not specifically address the effects of bead size, since the primary aim was to clarify the impact of T1DM.

Of note, no clear effects of different G content (Int G vs High G) were revealed, even with 46 blood donors. Nevertheless, Int G and High G differ in fibrosis outcomes in the murine model [36]; high G alginate induces a heightened fibrotic response compared to intermediate G alginate [34]. Fibrotic reactions to the biomaterials in general are orchestrated through foreign body responses (FBR), comprising chronological steps of protein adsorption and reactive proteins (complement and coagulation) as precursors of acute inflammation. At later stages, these responses might lead to chronic inflammation and foreign body giant cell (FBGC) formation and fibroblast growth [85,86]. The lack of differences in the current study may reflect some of the limitations of our *ex-vivo* whole blood model. Such short-term biocompatibility assays reflect the initial stages of protein absorption and, importantly, reactivities of complement/coagulation proteins that subsequently trigger inflammatory cytokine production [39,41,42]. However, the limited 4-h time window in whole blood may not adequately capture later events leading to chronic inflammation. The minimal differences observed between these microbeads in the blood model likely indicate their comparable activation potentials for complement and coagulation pathways. Thus, considering the *in vivo* effects, the G-content may impact other parts of the fibrotic process, such as monocyte-to-macrophage maturation, or other tissues and cells as endothelial cells and fibroblasts. Macrophages can be essential for fibrosis development in response to alginate microbeads [19]. In the study by Doloff et al., monocytes were recruited at early time points following implantation but were largely absent after 24 h, coinciding with the emergence of macrophages [19]. This temporal pattern suggests monocyte-to-macrophage maturation as part of the fibrotic response. We have previously demonstrated a connection between fibrinogen deposition patterns and fibrosis [36]. Coagulation could be an entrance point for fibrin formation; however, fibrin/fibrinogen deposition could also reflect simple adsorption of fibrinogen onto the material surface, given its high abundance in body fluids. More fibrinogen is deposited on High G versus Int G microbeads in mice [36]. Even though the current study revealed no clear difference in the immediate activation of coagulation and complement by High G versus Int G alginate microbeads, it is possible that the G content influences protein deposition patterns at later time-points *in vivo*.

Baseline plasma analysis revealed subtle differences in lipoproteins and metabolites in T1DM. It is well documented that T1DM patients have altered lipid metabolism, with glycemic control influencing lipoprotein profiles [87,88]. Poor glycemic control is linked to elevated triglycerides and LDL cholesterol, whereas well-managed T1DM typically presents with normal or decreased levels of LDL and normal or increased HDL [88,89]. Our study cohort, characterised by well-managed T1DM (HbA1c around 51 mmol/mol) and normal keto acids, showed higher HDL levels and relatively low LDL and VLDL levels. Peripheral hyperinsulinemia, a consequence of subcutaneous insulin delivery, may explain these findings by directly increasing lipoprotein lipase (LPL) activity. This reduces VLDL production and increases VLDL and LDL catabolism, resulting in reduced particle numbers [89]. By normalising particle numbers, we revealed a significant reduction in VLDL triglyceride (TG) content in T1DM, while smaller LDL subfractions exhibited a non-significant trend toward increased TG content. This finding is consistent with previous reports in well-controlled T1DM [89] and can also be attributed to the direct effects of insulin administration. Importantly, this alteration may contribute to the elevated cardiovascular disease (CVD) risk in T1DM [89]. However, functional changes in specific lipoprotein subclasses, such as cholesterol efflux capacity, antioxidative, anti-inflammatory, and anticoagulant activities, may be more critical determinants of CVD risk, which cannot be determined through quantitative or compositional analyses alone [89,90].

NMR analysis revealed a higher SPC signal in T1DM samples, likely reflecting higher HDL levels [61]. SPC levels have been reported to decrease during acute SARS-CoV-2 infection, and chronic inflammation [[91], [92], [93]]. Additionally, the glycosylated acute phase markers Glyc, GlycA and GlycB were slightly lower in T1DM, collectively suggesting a potentially lower basal inflammation state. However, given the lack of significant cytokine differences, the interpretation of these findings remains uncertain [94]. Human whole-blood models have proven valuable in evaluating biomaterial-related responses [44]. Our findings suggest that while different microbeads elicit distinct immune responses, whole blood responses in well-controlled T1DM closely resemble those in healthy controls. However, those results may not be generalisable to all T1DM patients eligible for islet transplantation. Nevertheless, whole blood models provide a useful platform for assessing early host responses to biomaterials intended for T1DM treatment. Despite their advantages, whole blood models have limitations in predicting long-term outcomes of transplantation, where dynamic changes in immune- and non-immune cell populations are central. Additionally, the presence of encapsulated islets in transplantation settings may introduce additional stress-related responses, necessitating further integrated studies.

The current data address the biocompatibility of implantable materials in participants with well-controlled type 1 diabetes mellitus (T1DM) and reveal no differences compared to matched healthy controls. This outcome answers our main question and could be positive regarding further developmental testing of materials for diabetes treatment. Moreover, the potential of sulfated alginate to reduce a broad range of inflammatory mediators further underscores its promise. Follow-up studies using animal models for diabetes treatment and fibrosis could be the next steps. A key question is why Int G and High G alginate microbeads are markedly different regarding fibrosis outcomes in mice models [36], yet affects the examined responses variable in blood to a similar degree. Future research should aim to elucidate the mechanisms underlying the differences in the mouse model, which have not yet been captured in the human *ex vivo* blood model. Proteomic profiling of implanted microcapsules and *in vitro* monocyte-to-macrophage maturation studies may aid in elucidating early protein adsorption dynamics and differences, contributing to the development of fibrosis-free materials [39,95]. Also, more detailed investigations into the role of alginate block compositions, modified through mannuronan C-5 epimerases, may provide valuable insights. Finally, a more detailed examination of how physicochemical characteristics, including size and surface charge, impact *ex vivo* whole blood inflammatory responses and *in vivo* fibrotic responses would be highly useful for tailoring materials with improved biocompatibility and therapeutic efficacy.

## 5 Conclusion

Despite underlying differences in lipoprotein metabolism between well-managed T1DM and healthy controls, *ex vivo* blood responses to alginate microspheres or immunostimulants were similar in both groups. Therefore, blood from healthy donors can serve as a relevant and practical approach for biocompatibility evaluations in the context of T1DM treatment. This study also highlights the material differences and potential benefits of sulfated alginates, particularly their immunomodulatory properties. These data have further potential use in integrated models for developing predictive models in optimised biomaterial design.

## CRediT authorship contribution statement

**Kalaiyarasi Vasuthas:** Writing – review & editing, Writing – original draft, Visualization, Methodology, Investigation, Formal analysis, Data curation. **Sverre Christian Christiansen:** Writing – review & editing, Resources. **Joachim Sebastian Kjesbu:** Writing – review & editing, Resources. **Liv Ryan:** Writing – review & editing, Methodology, Investigation. **Trygve Andreassen:** Writing – review & editing, Methodology, Investigation, Data curation. **Geir Slupphaug:** Writing – review & editing, Funding acquisition. **Berit L. Strand:** Writing – review & editing, Funding acquisition, Conceptualization. **Jørgen Stenvik:** Writing – review & editing, Writing – original draft, Supervision, Methodology, Investigation, Funding acquisition, Formal analysis, Data curation, Conceptualization. **Anne Mari A. Rokstad:** Writing – review & editing, Writing – original draft, Validation, Supervision, Resources, Project administration, Methodology, Investigation, Funding acquisition, Data curation, Conceptualization.

## AI usage

During the preparation of this work, the authors, as non-native English speakers, occasionally used Microsoft 365 Copilot to help refine the language. All content generated with the assistance of this tool was carefully reviewed and edited by the authors, who take full responsibility for the final publication.

## Funding

This study was supported by the 10.13039/100009123Norwegian University of Science and Technology (10.13039/100009123NTNU) health project and The Chicago Diabetes Project (www.chicagodiebetesproject.org), The Liaison Committee for Education, Research, and Innovation in Central Norway (Samarbeidsorganet) under grants 30398 (to GS) and 90162400 (to JS), The Joint Research Committee (FFU) grant 30406 (to JS), 10.13039/501100005416The Research Council of Norway (RCN) through its Centers of Excellence Funding Scheme, Grant 223255/F50 (to CEMIR), and Stiftelsen Biopolymer.

## Declaration of competing interest

The authors declare that they have no known competing financial interests or personal relationships that could have appeared to influence the work reported in this paper.

## Figures and Tables

**figs1:**
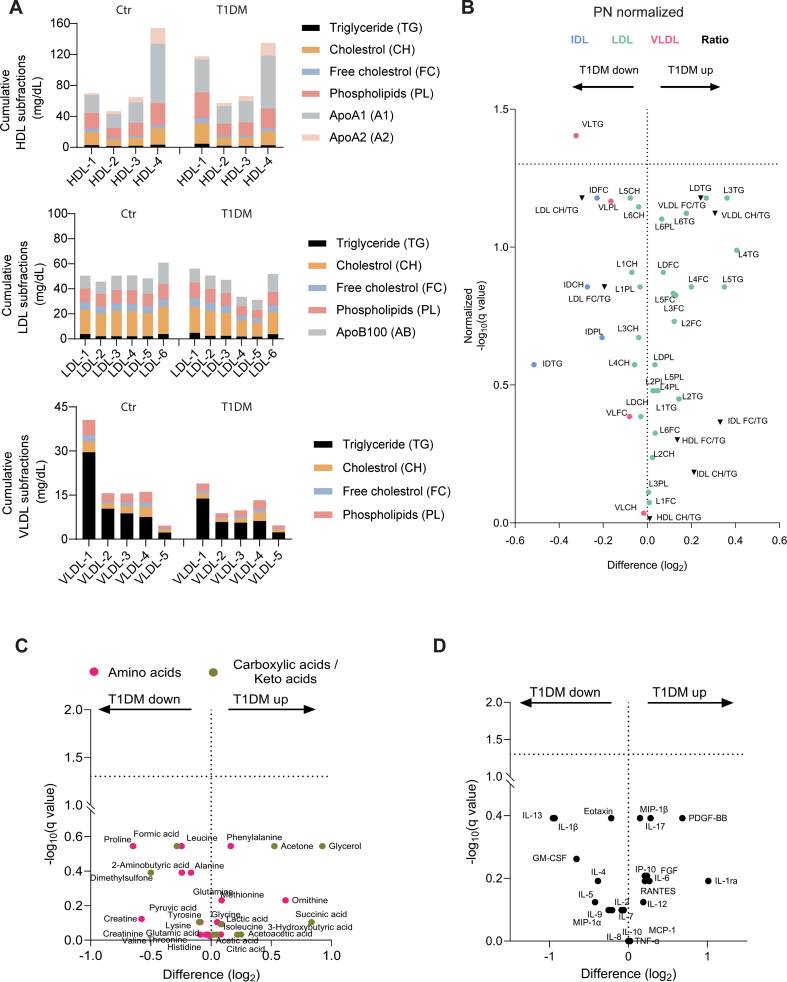


**figs2:**
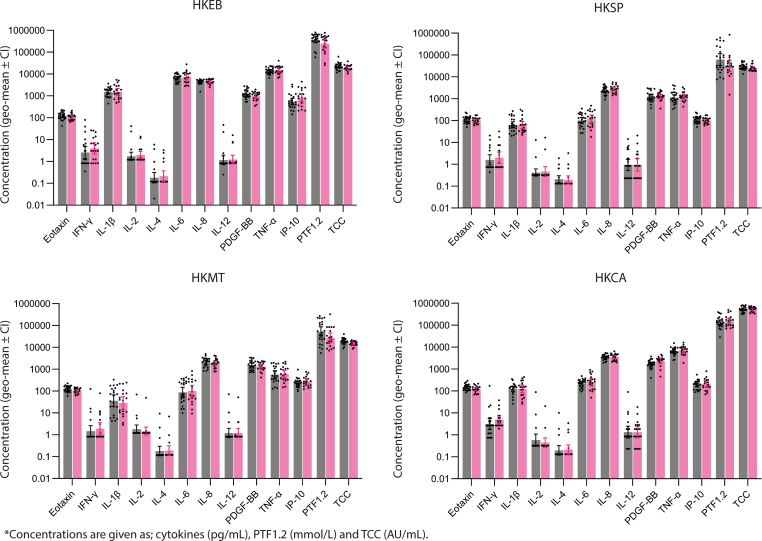


**figs3:**
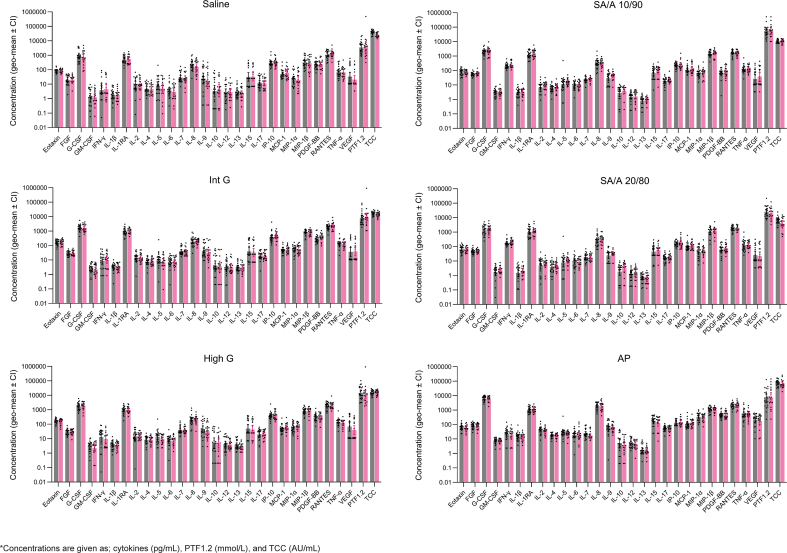


**figs4:**
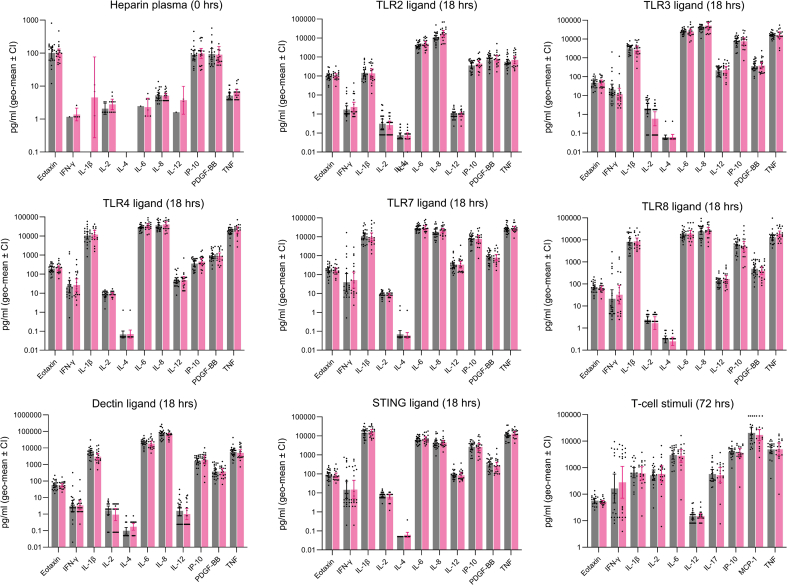


**figs5:**
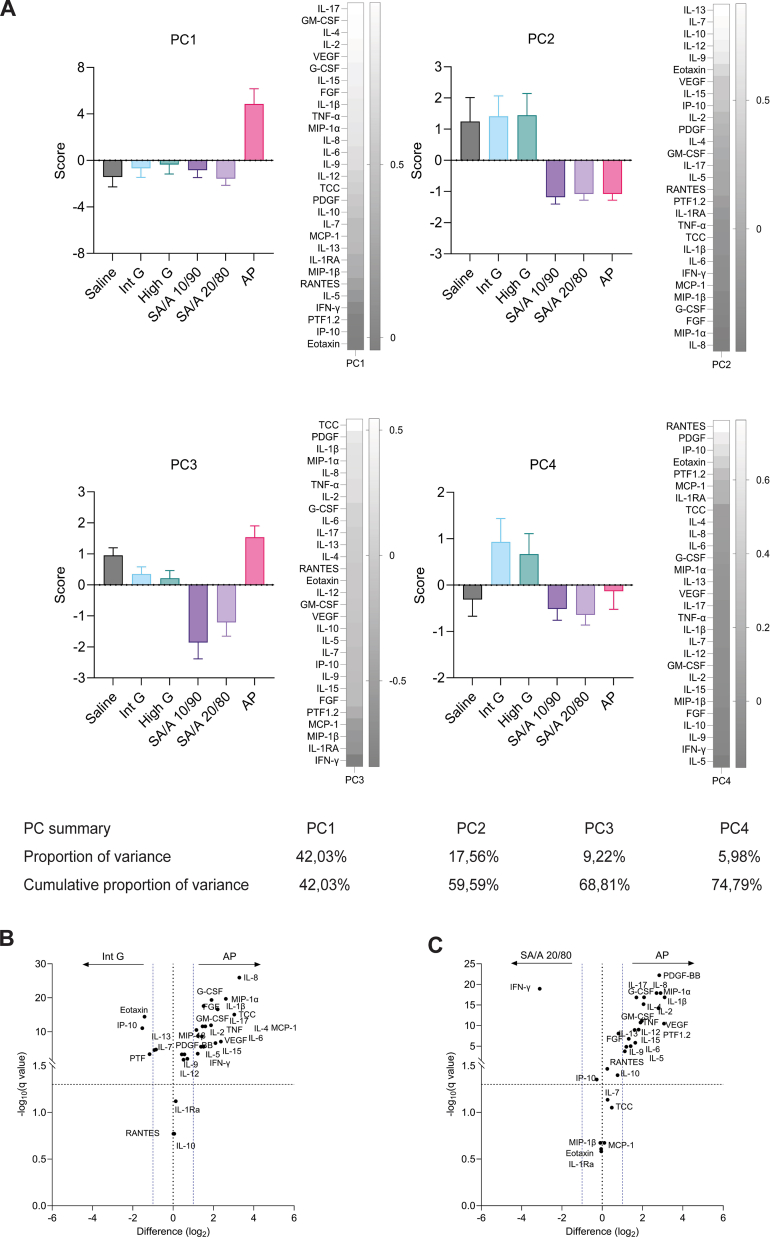


**figs6:**
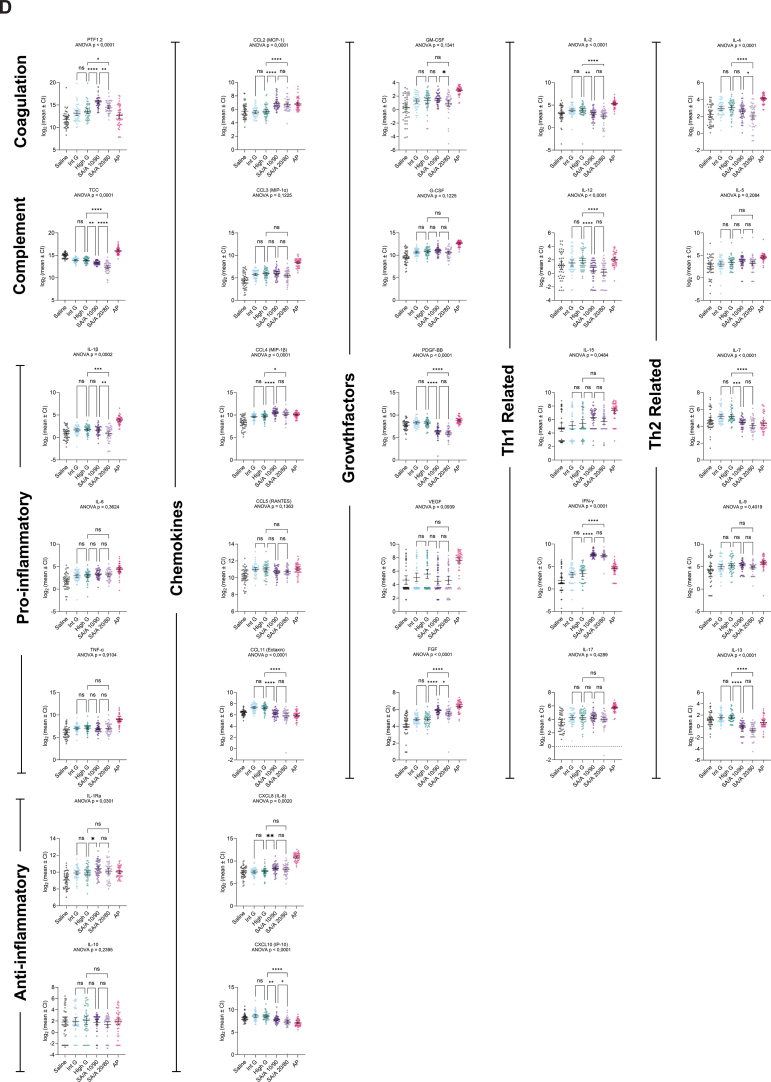


## Data Availability

The authors do not have permission to share data.

## References

[1] Katsarou A. (2017). Type 1 diabetes mellitus. Nat. Rev. Dis. Primers.

[2] Ganjali S. (2017). HDL functionality in type 1 diabetes. Atherosclerosis.

[3] Manjunatha S. (2016). Functional and proteomic alterations of plasma high density lipoproteins in type 1 diabetes mellitus. Metabolism.

[4] Cai Y. (2024). Lipid profile alterations and biomarker identification in type 1 diabetes mellitus patients under glycemic control. BMC Endocr. Disord..

[5] Zhang J. (2022). Lipid metabolism in type 1 diabetes mellitus: pathogenetic and therapeutic implications. Front. Immunol..

[6] Vergès B. (2024). Cardiovascular disease in type 1 diabetes, an underestimated danger: epidemiological and pathophysiological data. Atherosclerosis.

[7] Zarei M. (2025). Innovative immunotherapies and emerging treatments in type 1 diabetes management. Diabetes Epidemiology and Management.

[8] Schaaf C., Sussel L. (2025). A cure for type 1 diabetes: are we there yet?. Diabetes Technol. Therapeut..

[9] Kioulaphides S., García A.J. (2024). Encapsulation and immune protection for type 1 diabetes cell therapy. Adv. Drug Deliv. Rev..

[10] Qi M. (2012). Survival of human islets in microbeads containing high guluronic acid alginate crosslinked with Ca2+ and Ba2+. Xenotransplantation.

[11] Vegas A.J. (2016). Long-term glycemic control using polymer-encapsulated human stem cell–derived beta cells in immune-competent mice. Nat. Med..

[12] Schneider S. (2005). Long-term graft function of adult rat and human islets encapsulated in novel alginate-based microcapsules after transplantation in immunocompetent diabetic mice. Diabetes.

[13] Bochenek M.A. (2018). Alginate encapsulation as long-term immune protection of allogeneic pancreatic islet cells transplanted into the omental bursa of macaques. Nat. Biomed. Eng..

[14] Soon-Shiong P. (1994). Insulin independence in a type 1 diabetic patient after encapsulated islet transplantation. Lancet.

[15] Tuch B.E. (2009). Safety and viability of microencapsulated human islets transplanted into diabetic humans. Diabetes Care.

[16] Vaithilingam V., Bal S., Tuch B.E. (2017). Encapsulated islet transplantation: where do we stand?. Rev. Diabet. Stud..

[17] Kanak M.A. (2014). Inflammatory response in islet transplantation. Int J Endocrinol.

[18] Rokstad A.M. (2014). Advances in biocompatibility and physico-chemical characterization of microspheres for cell encapsulation. Adv. Drug Deliv. Rev..

[19] Doloff J.C. (2017). Colony stimulating factor-1 receptor is a central component of the foreign body response to biomaterial implants in rodents and non-human primates. Nat. Mater..

[20] Schreib C.C. (2023). Lipid deposition profiles influence foreign body responses. Adv Mater.

[21] Charlesworth J.A. (1987). The complement system in type 1 (insulin-dependent) diabetes. Diabetologia.

[22] Webb-Robertson B.M. (2024). Decrease in multiple complement proteins associated with development of islet autoimmunity and type 1 diabetes. iScience.

[23] de Paula Silva L. (2021). Prospection of plasma proteins as biomarkers for diabetes mellitus monitoring. J. Diabetes Metab. Disord..

[24] McMillan D.E. (1989). Increased levels of acute-phase serum proteins in diabetes. Metabolism.

[25] Oras A. (2019). A study of 51 subtypes of peripheral blood immune cells in newly diagnosed young type 1 diabetes patients. Clin. Exp. Immunol..

[26] Lin J. (2021). Analysis of immune cell components and immune-related gene expression profiles in peripheral blood of patients with type 1 diabetes mellitus. J. Transl. Med..

[27] Muhammad I.F. (2016). Acute-phase proteins and incidence of diabetes: a population-based cohort study. Acta Diabetol..

[28] Khamehchian T. (2017). Frequency of circulatory regulatory immune cells in Iranian patients with type 1 diabetes. Iran. J. Allergy Asthma Immunol..

[29] Deng C. (2015). Altered peripheral B-Lymphocyte subsets in type 1 diabetes and latent autoimmune diabetes in adults. Diabetes Care.

[30] Devaraj S. (2008). Increased toll-like receptor (TLR) 2 and TLR4 expression in monocytes from patients with type 1 diabetes: further evidence of a proinflammatory state. J. Clin. Endocrinol. Metab..

[31] Devaraj S. (2009). Increased levels of ligands of toll-like receptors 2 and 4 in type 1 diabetes. Diabetologia.

[32] Pradhan S., Brooks A.K., Yadavalli V.K. (2020). Nature-derived materials for the fabrication of functional biodevices. Mater. Today Bio.

[33] Grant G.T. (1973). Biological interactions between polysaccharides and divalent cations: the egg-box model. FEBS (Fed. Eur. Biochem. Soc.) Lett..

[34] Jacobs-Tulleneers-Thevissen D. (2013). Sustained function of alginate-encapsulated human islet cell implants in the peritoneal cavity of mice leading to a pilot study in a type 1 diabetic patient. Diabetologia.

[35] Dhawan A. (2020). Alginate microencapsulated human hepatocytes for the treatment of acute liver failure in children. J. Hepatol..

[36] Coron A.E. (2022). Pericapsular fibrotic overgrowth mitigated in immunocompetent mice through microbead formulations based on sulfated or intermediate G alginates. Acta Biomater..

[37] Liu Q. (2019). Zwitterionically modified alginates mitigate cellular overgrowth for cell encapsulation. Nat. Commun..

[38] Arlov Ø. (2014). Heparin-Like properties of sulfated alginates with defined sequences and sulfation degrees. Biomacromolecules.

[39] Vasuthas K. (2025). Fucoidan alginate and sulfated alginate microbeads induce distinct coagulation, inflammatory and fibrotic responses. Mater. Today Bio.

[40] Rokstad A.M. (2011). Alginate microbeads are complement compatible, in contrast to polycation containing microcapsules, as revealed in a human whole blood model. Acta Biomater..

[41] Rokstad A.M. (2013). The induction of cytokines by polycation containing microspheres by a complement dependent mechanism. Biomaterials.

[42] Ørning P. (2016). Alginate microsphere compositions dictate different mechanisms of complement activation with consequences for cytokine release and leukocyte activation. J Control Release.

[43] Gravastrand C.S. (2019). Cholesterol crystals induce coagulation activation through complement-dependent expression of monocytic tissue factor. J. Immunol..

[44] Rokstad A.M. (2013). Biocompatibility and biotolerability assessment of microspheres using a whole blood model. Micro Nanosyst..

[45] King A., Sandler S., Andersson A. (2001). The effect of host factors and capsule composition on the cellular overgrowth on implanted alginate capsules. J. Biomed. Mater. Res..

[46] Strand B.L. (2001). Poly-L-Lysine induces fibrosis on alginate microcapsules via the induction of cytokines. Cell Transplant..

[47] Arlov Ø., Skjåk-Bræk G. (2017). Sulfated alginates as heparin analogues: a review of chemical and functional properties. Molecules.

[48] Grasdalen H., Larsen B., Smidsrød O. (1979). A p.m.r. study of the composition and sequence of uronate residues in alginates. Carbohydr. Res..

[49] Grasdalen H. (1983). High-field, 1H-n.m.r. spectroscopy of alginate: sequential structure and linkage conformations. Carbohydr. Res..

[50] Vold I.M.N., Kristiansen K.A., Christensen B.E. (2006). A study of the chain stiffness and extension of alginates, in vitro epimerized alginates, and periodate-oxidized alginates using size-exclusion chromatography combined with light scattering and viscosity detectors. Biomacromolecules.

[51] Mørch Y.A. (2006). Effect of Ca2+, Ba2+, and Sr2+ on alginate microbeads. Biomacromolecules.

[52] Bruker B.I. Methods*.*; Available from: https://www.bruker.com/en/products-and-solutions/mr/nmr-clinical-research-solutions/b-i-methods.html.

[53] Bruker B.I. Methods (PACS). Available from: https://www.bruker.com/en/products-and-solutions/mr/nmr-clinical-research-solutions/phenorisk-pacs.html.

[54] B.I.Quant-PS. Available from: https://www.bruker.com/en/products-and-solutions/mr/nmr-clinical-research-solutions/b-i-quant-ps.html.

[55] B.I.LISA. Available from: https://www.bruker.com/en/products-and-solutions/mr/nmr-clinical-research-solutions/b-i-lisa.html.

[56] Mollnes T.E. (2002). Essential role of the C5a receptor in E coli-induced oxidative burst and phagocytosis revealed by a novel lepirudin-based human whole blood model of inflammation. Blood.

[57] Gravastrand C. (2017). Alginate microbeads are coagulation compatible, while alginate microcapsules activate coagulation secondary to complement or directly through FXII. Acta Biomater..

[58] Mouton W. (2020). Towards standardization of immune functional assays. Clin. Immunol..

[59] Connelly M.A. (2017). GlycA, a novel biomarker of systemic inflammation and cardiovascular disease risk. J. Transl. Med..

[60] Otvos J.D. (2015). GlycA: a composite nuclear magnetic resonance biomarker of systemic inflammation. Clin. Chem..

[61] Masuda R. (2022). Exploration of human serum lipoprotein supramolecular phospholipids using statistical heterospectroscopy in n-Dimensions (SHY-n): identification of potential cardiovascular risk biomarkers related to SARS-CoV-2 infection. Anal. Chem..

[62] Landsem A. (2015). The key roles of complement and tissue factor in escherichia coli-induced coagulation in human whole blood. Clin. Exp. Immunol..

[63] Gil M.L., Gozalbo D. (2009). Role of toll-like receptors in systemic Candida albicans infections. Front. Biosci. (Landmark Ed.).

[64] Cejkova P. (2016). TLR2 and TLR4 expression on CD14(++) and CD14(+) monocyte subtypes in adult-onset autoimmune diabetes. Biomed Pap Med Fac Univ Palacky Olomouc Czech Repub.

[65] Zahran A.M. (2021). Analysis of toll-like Receptor-2 and 4 expressions in peripheral monocyte subsets in patients with type 1 diabetes mellitus. Immunol. Investig..

[66] Kurianowicz K. (2021). Impaired innate immunity in pediatric patients type 1 diabetes—focus on toll-like receptors expression. Int. J. Mol. Sci..

[67] Han D. (2012). Innate and adaptive immune gene expression profiles as biomarkers in human type 1 diabetes. Clin. Exp. Immunol..

[68] Wen L. (2004). The effect of innate immunity on autoimmune diabetes and the expression of toll-like receptors on pancreatic islets. J. Immunol..

[69] Janssen A.W.M. (2021). Understanding the increased risk of infections in diabetes: innate and adaptive immune responses in type 1 diabetes. Metabolism.

[70] Eyerich K. (2008). Patients with chronic mucocutaneous candidiasis exhibit reduced production of Th17-Associated cytokines IL-17 and IL-22. J. Invest. Dermatol..

[71] Duffy D. (2014). Functional analysis via standardized whole-blood stimulation systems defines the boundaries of a healthy immune response to complex stimuli. Immunity.

[72] Arlov Ø., Skjåk-Bræk G., Rokstad A.M. (2016). Sulfated alginate microspheres associate with factor H and dampen the inflammatory cytokine response. Acta Biomater..

[73] Luque-Martin R. (2021). IFN-γ drives human monocyte differentiation into highly proinflammatory macrophages that resemble a phenotype relevant to psoriasis. J. Immunol..

[74] Kang K. (2019). IFN-γ selectively suppresses a subset of TLR4-activated genes and enhancers to potentiate macrophage activation. Nat. Commun..

[75] Italiani P. (2014). From monocytes to M1/M2 macrophages: phenotypical vs. Functional differentiation. Front. Immunol..

[76] Valente A.J. (1998). A complex element regulates IFN-gamma-stimulated monocyte chemoattractant protein-1 gene transcription. J. Immunol..

[77] Li Q. (2017). The heparin-like activities of negatively charged derivatives of low-molecular-weight polymannuronate and polyguluronate. Carbohydr. Polym..

[78] Coron A.E. (2022). MS-proteomics provides insight into the host responses towards alginate microspheres. Mater. Today Bio.

[79] Dorchei F. (2024). Postmodification with polycations enhances key properties of alginate-based multicomponent microcapsules. Biomacromolecules.

[80] de Vos P. (2007). Zeta-potentials of alginate-PLL capsules: a predictive measure for biocompatibility?. J. Biomed. Mater. Res..

[81] Tam S.K. (2005). Physicochemical model of alginate-poly-L-lysine microcapsules defined at the micrometric/nanometric scale using ATR-FTIR, XPS, and ToF-SIMS. Biomaterials.

[82] van Hoogmoed C.G., Busscher H.J., de Vos P. (2003). Fourier transform infrared spectroscopy studies of alginate-PLL capsules with varying compositions. J. Biomed. Mater. Res..

[83] Ashimova A. (2019). Cell encapsulation within alginate microcapsules: immunological challenges and outlook. Front. Bioeng. Biotechnol..

[84] Veiseh O. (2015). Size- and shape-dependent foreign body immune response to materials implanted in rodents and non-human Primates. Nat. Mater..

[85] Zhou X. (2024). Materials strategies to overcome the foreign body response. Adv. Healthcare Mater..

[86] Liu L. (2025). Advances in the application and research of biomaterials in promoting bone repair and regeneration through immune modulation. Mater. Today Bio.

[87] Hughes T.A. (2016). Lipoprotein composition in patients with type 1 diabetes mellitus: impact of lipases and adipokines. J Diabetes Complications.

[88] Vergès B. (2009). Lipid disorders in type 1 diabetes. Diabetes & metabolism.

[89] Vergès B. (2020). Dyslipidemia in type 1 diabetes: a masked danger. Trends in Endocrinology & Metabolism.

[90] Camont L., Chapman M.J., Kontush A. (2011). Biological activities of HDL subpopulations and their relevance to cardiovascular disease. Trends Mol. Med..

[91] Lodge S. (2021). Diffusion and relaxation edited proton NMR spectroscopy of plasma reveals a high-fidelity supramolecular biomarker signature of SARS-CoV-2 infection. Anal. Chem..

[92] Stadler J.T. (2023). HDL-related parameters and COVID-19 mortality: the importance of HDL function. Antioxidants.

[93] Kazenwadel J. (2023). Stratification of hypertension and SARS-CoV-2 infection by quantitative NMR spectroscopy of human blood serum. Commun. Med..

[94] Syed Khaja A.S. (2024). Clinical importance of cytokine (IL-6, IL-8, and IL-10) and vitamin D levels among patients with Type-1 diabetes. Sci. Rep..

[95] Stewart C.L. (2024). Cellular and microenvironmental cues that promote macrophage fusion and foreign body response. Front. Immunol..

