# Supplementary material for: Type 1 diabetes mellitus (T1DM) does not affect whole blood responses to alginate-based microspheres despite plasma lipid and glucose differences
Source: Mater Today Bio. 2025 Jul 18;34:102113. doi: 10.1016/j.mtbio.2025.102113 (PMC12318296; doi:10.1016/j.mtbio.2025.102113)
Supplement: Multimedia component 1 [file mmc1.docx]

**Supplementary figure captions**

***Fig. S1. Baseline plasma levels of lipoproteins, metabolites and cytokines in T1DM (n = 19) and healthy controls (n = 22). (A)*** *Composition of lipoprotein subclasses in healthy (left) and T1DM (right) subjects.* ***(B)*** *Comparison of IDL, LDL and VLDL lipoproteins molecular composition in T1DM versus healthy controls after normalising by the particle numbers* ***(C)*** *Comparison of plasma amino acids and carboxylic/keto acids concentration in the groups.* ***(D)*** *Plasma cytokine level comparison across the groups.*

***Fig. S2. Levels of individual cytokines, TCC, and PTF.1.2 following incubation with different alginate microspheres.*** *T1DM (pink bars, n = 19) and healthy controls (grey bars, n = 22). Concentrations are given as: cytokines (pg/mL), PTF1.2 (mmol/L), and TCC (AU/mL).*

***Fig. S3: Levels of individual cytokines, TCC, and PTF.1.2 following incubation with bioparticles (HKEB, HKSP, HKMT and HKCA).*** *T1DM (pink bars, n = 19, for IP-10 n = 18) and healthy controls (grey bars, n = 22). Concentrations are given as: cytokines (pg/mL), PTF1.2 (mmol/L), and TCC (AU/mL).*

***Fig. S4: Levels of individual cytokines, TCC, and PTF.1.2 following incubation with distinct TLR ligands (TLR2, TLR3, TLR4, TLR7, TLR8) ligands for Dectin and STING, and a T cell stimulus.*** *Bar plots show the comparison of variables between T1DM (pink bars, n = 19) and healthy controls (grey bars, n = 19). Concentrations are given as: cytokines (pg/mL), PTF1.2 (mmol/L), and TCC (AU/mL).*

***Fig. S5: PCA of immune responses induced in whole blood by treatment with saline and the microcapsules. (A)*** *Bar plots of the selected PC (PC1-PC4) score values and the associated heatmap of the variables loading per PC. Variance coverage of each PC is shown in the chart below. The volcano plot from the direct comparison of the responses to* ***(B)*** *Int G vs AP* ***(C)*** *SA/A 20/80 vs AP.* ***(D)*** *Quantification of all variables after treatment. Overall ANOVA p-values above 0.0002 and post-test p-values from the pairwise comparisons are indicated*. *Significant values are given as p ≤ 0.05 (*), p ≤ 0.01 (**), p ≤ 0.001 (***), and p ≤ 0.0001 (****) compared to selected microspheres.*
